# Text Analysis of Radiology Reports with Signs of Intracranial Hemorrhage on Brain CT Scans Using the Decision Tree Algorithm

**DOI:** 10.17691/stm2022.14.6.04

**Published:** 2022-11-28

**Authors:** А.N. Khoruzhaya, D.V. Kozlov, К.M. Arzamasov, E.I. Kremneva

**Affiliations:** Junior Researcher, Department of Innovative Technologies; Research and Practical Clinical Center for Diagnostics and Telemedicine Technologies of the Moscow Health Care Department, Bldg 1, 24 Petrovka St., Moscow, 127051, Russia;; Junior Researcher, Department of Medical Informatics, Radiomics and Radiogenomics; Research and Practical Clinical Center for Diagnostics and Telemedicine Technologies of the Moscow Health Care Department, Bldg 1, 24 Petrovka St., Moscow, 127051, Russia;; Head of the Department of Medical Informatics, Radiomics and Radiogenomics; Research and Practical Clinical Center for Diagnostics and Telemedicine Technologies of the Moscow Health Care Department, Bldg 1, 24 Petrovka St., Moscow, 127051, Russia;; Leading Researcher, Department of Innovative Thechnologies; Research and Practical Clinical Center for Diagnostics and Telemedicine Technologies of the Moscow Health Care Department, Bldg 1, 24 Petrovka St., Moscow, 127051, Russia; Senior Researcher; Research Center of Neurology, 80 Volokolamskoye Shosse, Moscow, 125367, Russia

**Keywords:** computed tomography, diagnostic reports, intracranial hemorrhage, natural language processing, machine learning, decision tree algorithm

## Abstract

**The aim of the study** is to create, train, and test the algorithm for the analysis of brain CT text reports using a decision tree model to solve the task of simple binary classification of presence/absence of intracranial hemorrhage (ICH) signs.

**Materials and Methods:**

The initial data is a download from the Unified Radiological Information Service of the Unified Medical Information and Analytical System (URIS UMIAS) containing 34,188 studies obtained by a non-contrast CT of the brain in 56 inpatient medical settings. Data analysis and preprocessing were carried out using NLTK (Natural Language Toolkit, version 3.6.5), a library for symbolic and statistical processing of natural language, and scikit-learn, a machine learning library containing tools for classification tasks. According to 14 selected ICH-related key words, as well as 33 stop-phrases with key words denoting absence of ICH, an automatic selection of the CT investigations and their subsequent expert verification were carried out. Two classes of investigations were formed based on the sample from 3980 protocol descriptions: containing descriptions of ICH and without them. The problem of binary classification was solved using the decision tree algorithm as a model. To evaluate the performance of the model, the CT investigations were divided randomly into samples in the ratio of 7:3. Of 3980 protocols, 2786 were assigned to the training data set, 1194 — to the test one.

**Results:**

According to the test results, the designed and trained algorithm in the binary classification of the CT reports “with signs of ICH” and “without signs of ICH” has shown sensitivity of 0.94, specificity of 0.88, F-score of 0.83.

**Conclusion:**

The developed and trained algorithm for the analysis of radiology reports has demonstrated high accuracy in relation to brain CT with signs of intracranial hemorrhage and can be used to solve binary classification problems and create appropriate data sets. However, it is limited by the need for manual revision of CT studies to ensure quality control.

## Introduction

Modern healthcare settings generate and accumulate large volumes of information of various categories: text records of medical patients’ cards with the description of complaints and case histories, referrals to examinations, summary reports, text and digital results of laboratory and instrumental investigations, digital medical images, and so on. The main bulk of this information falls on unstructured text medical data. Nevertheless, in the majority of cases they contain the most valuable information, which may be the basis for developing new tools of digital medicine: medical decision support systems, various electronic medical assistants, models of predicting disease development, and other attributes of ongoing digital healthcare transformation [1].

The number of diagnostic examination reports generated during a year (for example, computed tomograms) amounts to more than a hundred thousand and grows with each year. Thus, according to the data of Unified Radiological Information Service of the Unified Medical Information and Analytical System (URIS UMIAS), 111,487 CT investigations were conducted in the outpatient polyclinic settings of the Moscow Department of Health for 9 months in 2017 [2], while in 2021 their quantity increased already to 777,402. Timely retrieval of the necessary information obtained by radiological examinations and its subsequent analysis by means of machine learning algorithms may facilitate fast and effective decisions in the diagnosis of different pathologies and improve the quality of the appropriate medical aid. It is especially important in the sphere of emergency care, for instance, in timely diagnosis of intracranial hemorrhages (ICH) [3-5].

The development of qualitative machine learning algorithms for the analysis of medical images demands qualitative data sets. Initial selection of such data from the whole array of investigations may be done manually, which requires much time, but it may also be automated on the basis of the analysis of unstructured text protocols of radiological records. Methods of natural language processing (NLP) transform this unstructured text into a structured form, from which it is possible to extract information carrying the necessary semantic load [6]. Thus, automatic processing of protocol descriptions allows one to select radiological examinations with the desired signs.

The efficiency of machine learning depends in many respects on how well the data are labeled in the training sample, which in its turn requires much effort of the medical experts possessing highly specialized knowledge. To reduce the time spent on this task and simplify the process of labeling and not to worsen the outcome, there exists the strategy of a weak supervision for the algorithms of machine learning on the weakly labeled training data [7]. It has found a wide application in the fields of biomedicine specifically for the classification tasks [8, 9]. The idea of it is as follows: at the initial stage of the algorithm training, weak labels are created which are analyzed by a medical expert. In this way, a data set is formed, which is used for further training. Finally, a trained model is obtained for extracting information from the unstructured clinical text. The proposed scheme was evaluated in the tasks of binary classification and has demonstrated high operation accuracy up to 0.97 [10].

Simple models with the possibility of automatic learning should be chosen for these tasks. One of such models combining these features is the algorithm of a decision tree [11]. Besides, interpretability of the model seems to be an important factor, and the decision tree algorithm is found to have the highest interpretability [12]. Therefore, we have chosen its application in our work.

In the literature, there are little data on the efficiency of decision tree algorithms for classification of medical texts and the protocols describing CT of the brain investigations in particular. The developments in classification of the texts in foreign languages, e.g. English [13] and Chinese [14], are presented more widely, but there are actually no papers analyzing Russian medical texts. At the same time, the solution of this task would allow one to analyze a stream of diagnostic investigations by the frequency of pathology occurrence, and to sample investigations for preparation and quality control of the datasets when training computer vision algorithms designed for the analysis of medical images, although application of these algorithms in the diagnostic radiology is not limited by these tasks [15].

**The aim of the study** is to create, train, and test the algorithm for the analysis of brain CT text reports using a decision tree model to solve the task of simple binary classification of presence/absence of intracranial hemorrhage signs.

## Materials and Methods

The initial data is a download from the URIS UMIAS containing 34,188 studies obtained as a result of non-contrast CT of the brain in 56 inpatient medical settings. Each line of this dataset contains the following information: unique identifier, age, sex, date of diagnosis, a list of medical institutions participating in the investigation, and the record and summary of the investigation.

The following factors served as criteria for exclusion of CT investigations from the sample: absence of protocols with descriptions and medical conclusions (empty fields in these lines), age under 18 years, absence of information about age or its abnormal value due to the incorrect date input (976, 1000 years), complete line duplicates. For this reason, the number of investigations whose data were included into the sample was equal to 29,682. Accordingly, completed fields with the text records and medical conclusions, absence of abnormal patients’ age, and duplication of information became criteria for inclusion in the sample.

All investigations were performed in the period from 12 A.M. January 1, 2020 to 12 A.M. December 31, 2020. There were 14,895 women and 14,787 men. The minimal age was 18 and maximal was 99 years.

The considered task of evaluating ICH presence according to the results of the text protocols describing CT brain investigations irrespective of localization of their conductance represents a task of binary classification: absence/presence of hemorrhage. The analysis of data and their preprocessing was done using the NLTK library (Natural Language Toolkit, v. 3.6.5) for symbolic and statistical processing of natural language, and scikit-learn, a machine learning library containing tools for classification tasks. These libraries and subsequent algorithm were written using Python (v. 3.9.7) programming language.

**Figure 1.**
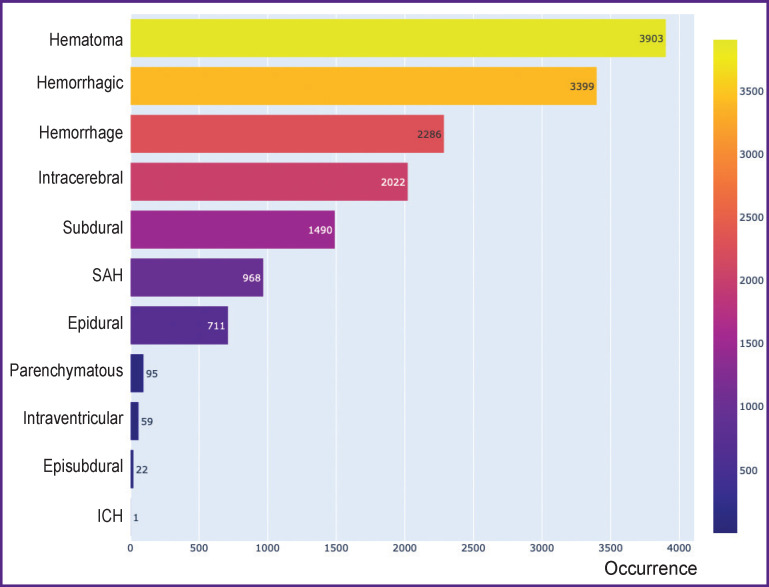
Distribution of frequency of occurrence (*horizontal axis*) of the key words (*vertical axis*) related to intracranial hemorrhage in the initial sample Here: ICH — intracerebral hemorrhage; SAH — subarchnoid hemorrhage

An initial sample for machine learning was presented by the CT investigations containing 14 ICH-related key words in the record and medical conclusions: hemorrhage(s), hematoma(s), hemorrhagic, intracerebral, subarachnoid, epidural, subdural, intraventricular, parenchymatous, episubdural; SAH (subarachnoid hemorrhage), EDH (epidural hemorrhage), SDH (subdural hemorrhage), ICH (intracerebral hemorrhage). These key words were chosen based on the expert opinion of the radiologist working over 3 years in this field. The distribution of the key words in the initial sample is shown in Figure 1.

**Figure 2.**
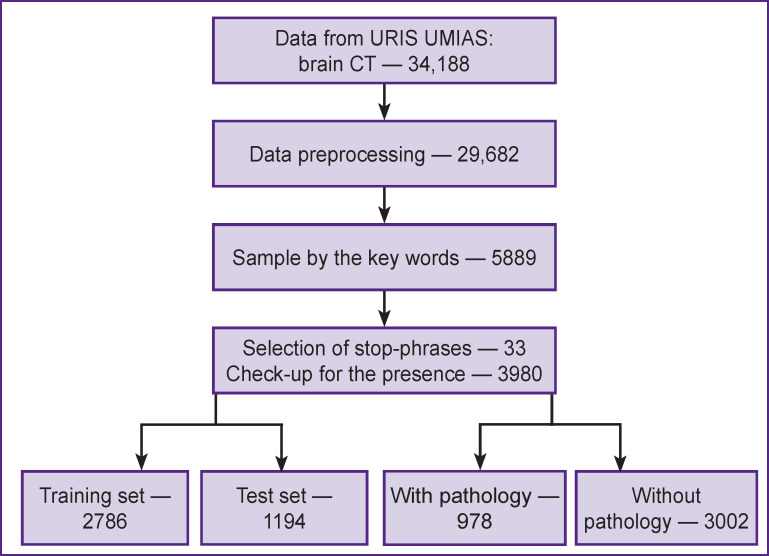
Study design

At this stage, selection was done “mechanically”, i.e. by the presence of this key (word) in the text not considering the surrounding words. The number of CT investigations amounted to 5889 after this stage (Figure 2).

However, selection by key words was not the solution of the task for us. The fact is that a key word in combination with negation (stop-word or stop-phrase) means absence of the target pathology. Radiologists often use similar stop-phrases in the protocols describing CT investigations. Therefore, search for the key words only does not allow correct acquisition of the necessary data. As a result, a sample (5889) generated in this way was verified by three radiologists with work experience over 3 years. They helped compose a list of 33 stop-phrases, the content of which in the protocol implied absence of any ICH in the investigation. Examples of some of stop-phrases are given below:

foci of pathological density of the brain matter are not found;

CT data for intracranial hematoma and brain contusion are not obtained;

signs of intracranial hemorrhage are not found;

in the obtained images, foci of pathological density in the brain matter are not determined;

CT signs of intracranial hematoma, cranial bone fractures, other focal and volumetric brain matter alterations are not detected.

**Figure 3.**
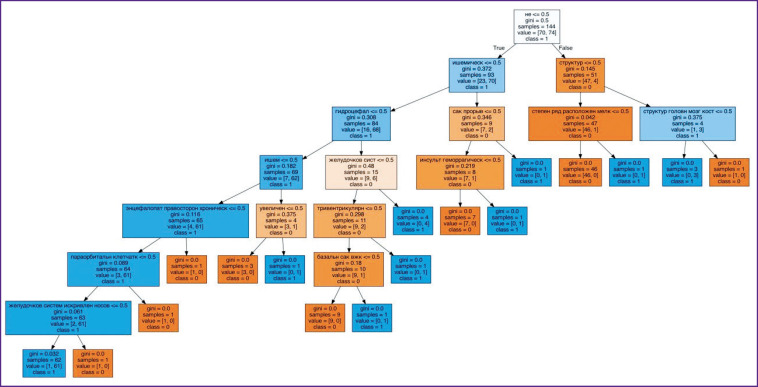
The developed algorithm for the analysis of text descriptions of brain CT investigations with the signs of intracranial hemorrhages

At the next stage, automatic selection of CT investigations, in which the key words and stop-phrases were present, was repeated. The finally obtained 3980 investigations were divided in two classes: containing ICH reports (978) and without them (3002). The algorithm of a decision tree was chosen as a model for solving the task of binary classification. DecisionTreeClassifier is one of the classification methods in machine learning using the scikit-learn library. The maximal depth of the decision tree was selected empirically and was equal to 15 levels. The algorithm operating quality was evaluated using the classification_ report function (Figure 3).

To assess the performance of the model, the CT investigations were randomly divided into samples in the ratio of 7:3, since it is this ratio of the training/testing set of data allows one to obtain the most optimal metrics of the algorithm performance [16]. Of 3980 protocols, 2786 were assigned to the training data set, 1194 to the test one. Of 1194 test sets, 927 did not contain the signs of ICH, 267 had these signs.

## Results and Discussion

**Figure 4.**
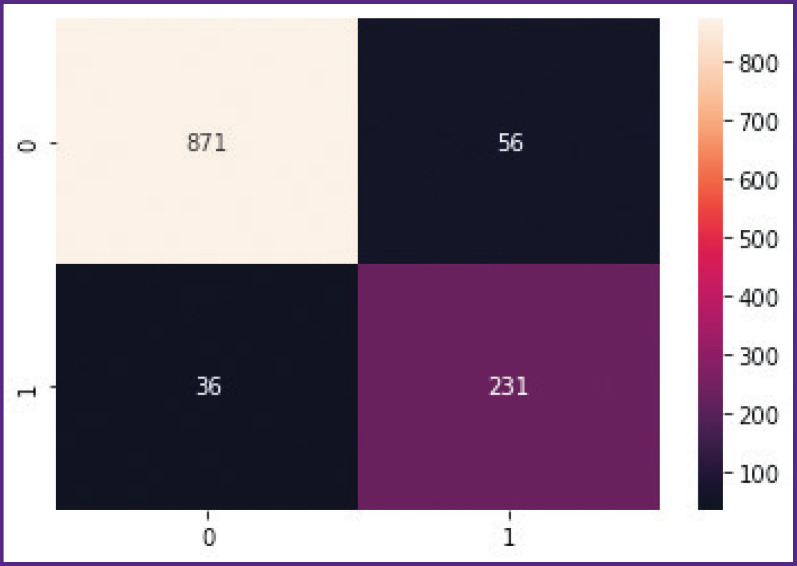
Error matrix: vertically — true estimate of CT investigation: 0 — investigation without signs of intracerebral hemorrhages (true-negative result); 1 — investigation with signs of hemorrhages (true-positive result); horizontally — estimates using the developed algorithm: 0 — presence of pathology was found erroneously (false-positive result); 1 — absence of pathology was indicated erroneously (false-negative result)

According to the testing results, the trained algorithm in the binary classification of the text protocols of CT investigations “with ICH signs” and “without ICH signs” has demonstrated sensitivity of 0.94 (95% CI: 0.942– 0.939) and specificity of 0.88 (95% CI: 0.841–0.919). Positive prognostic significance was 0.96, i.e. the CT investigation with the label “pathology” selected by our algorithm will have its feature with the probability of 96%. In its turn, the negative prognostic significance appeared to be 0.81, consequently, the algorithm will give the correct reply about the absence of pathological signs in the text, where they are really absent, with the probability of 81%. The F-score was equal to 0.83. This parameter represents a weighted harmonic mean and unites the recall and precision of the tested algorithm. To make the obtained results more clear, a fourfold table, the error matrix, is presented in Figure 4.

The data obtained in the process of our study are quite in line with those presented in the literature. Thus, in the work by Hostettler et al. [12], application of the methods of NLP has shown sufficiently high efficiency in identification and outcome of diseases. The clinical outcome in subarachnoid hemorrhage on days 1, 3, and 7 was predicted using the data of laboratory investigations of 548 patients with the help of decision tree algorithm. The model had the highest accuracy for the first day. Sensitivity for lethal outcome prediction was 83.1%, specificity was 75.3%. However, it should be noted that in this work, the authors analyzed the laboratory indices, presentation of which possessed a high degree of standardization.

Based on the unstructured protocols of diagnostic reports, Warner et al. [17] evaluated the accuracy of predicting lung cancer with the help of decision tree algorithm. The analysis by machine learning methods allowed them to classify 751,880 medical text-based documents from 2327 patients. Despite the availability of large variants of reports in the documentation, the accuracy of the lung cancer stage estimated by the algorithm appeared to be rather high — 0.906 (95% CI: 0.873–0.939).

Szlosek and Ferretti [18] considered the possibility of using machine learning algorithms for NLP to automate the assessment of clinical decision support systems in electronic medical record systems. The set of data contained information about the results of CT brain investigations of 3621 patients with a mild traumatic brain injury. The classifier built on the basis of the decision tree algorithm has demonstrated sensitivity of 57.75% and a much higher specificity of 98.68%.

The algorithm developed by us also showed a slightly lower sensitivity since there was a larger number of false-positive estimates than false-negative ones. It was mainly due to the use of both stop-phrases and descriptions of extracranial pathology using key words in the protocols of CT investigations. For example, the stop-phrase “fresh hemorrhages or ischemic changes in the brain were not detected” was written in the text protocol together with the description of hemorrhage into the facial soft tissues. Nevertheless, the sensitivity values obtained by us are higher than those presented in the literature.

False-negative estimates of the algorithm are associated with the “conflict” of the simultaneously present stop-phrase in the protocol, which is interpreted by the algorithm as a feature of pathology absence (for example, “data on intracranial hematoma in this investigation was not found”) and key words used to describe, for instance, a small zone of hemorrhagic impregnation of the brain tissues.

The main problem related to using the tools of NPL methods based on machine learning, with the application of the decision tree algorithms, in particular, lies in the absence of report standardization. Machine learning of this kind implies simple classification, and the type of the data which underwent unification is suitable for its successful application, for example, digital values of laboratory studies, or the categories in the BI-RADS system for analyzing and reporting the results of radiological breast examination [19].

The diagnostic accuracy metrics obtained in the present study indicates the possibility of practical application of the developed algorithm in accordance with the requirements of the methodological recommendations “Clinical trials of software based on intelligent technologies (diagnostic radiology)” [20].

This algorithm may be employed at the first stage of preparation of data sets for initial, rough selection of CT investigations with the necessary features from a large array of information, for example, from the direct download of all CT investigations of the brain over a year. Then the set of CT investigations may be further analyzed, for example, by means of neural networks for a finer selection by the features (if you need only cases without surgical intervention, or CT scans with a specific type of hemorrhage). Initial datasets may be also used for training or testing diagnostic services based on artificial intelligence.

The developed algorithm may be successfully used at the stage after rendering a medical aid in any type of medical settings to control the quality of doctors’ work and simplify the preparation of statistical reports.

However, there are some restrictions of the work presented here. Presently, it is a pilot version for classification of brain CT text reports with ICH signs. The following drawbacks should be noted in the developed decision tree algorithm: false estimates, difficulties with classification of unstructured text having multiple variations of descriptive meanings of presence and absence of pathology, the necessity of manual revision of investigations to ensure quality control. These disadvantages point to need of complicating the classifier and using other approaches of machine learning including neural networks.

## Conclusion

The developed and trained algorithm for the analysis of radiology text reports based on the decision tree model has demonstrated high accuracy in selection of brain CT investigations with the signs of intracranial hemorrhage. It can be used to solve binary classification tasks and to optimize creation of appropriate sets of diagnostic studies, which will be used for training, and validation of medical services on the basis of artificial intelligence directed to the diagnosis of hemorrhages according to the brain CT investigations. Besides, after the appropriate training, it may be employed for the analysis and binary classification of any other medical texts, as well as for the supervision of diagnosis and medical aid.
